# Interactions of Spring Cereal Genotypic Attributes and Recovery of Grain Yield After Defoliation

**DOI:** 10.3389/fpls.2020.00607

**Published:** 2020-06-05

**Authors:** Lindsay W. Bell, John A. Kirkegaard, Lihua Tian, Sally Morris, John Lawrence

**Affiliations:** ^1^CSIRO Agriculture and Food, Toowoomba, QLD, Australia; ^2^CSIRO Agriculture and Food, Canberra, ACT, Australia; ^3^College of Pastoral Agriculture Science and Technology, Lanzhou University, Lanzhou, China; ^4^School of Biosciences, University of Nottingham, Loughborough, United Kingdom

**Keywords:** regrowth, radiation interception, yield components, tillering, grazing, soluble carbohydrates

## Abstract

Dual-purpose crops are grazed during their vegetative phase and allowed to regrow to produce grain. Grazing slow-developing winter cereals (wheat, barley, and triticale) is common, but there is also potential to graze faster-developing spring cereals used in regions with shorter-growing seasons. Defoliation in faster-developing genotypes has risks of larger yield penalties, however, little is known about genotypic characteristics that may improve recovery after grazing. Four experiments examined 7 spring wheat and 2 barley cultivars with differing physiological attributes (phenological development rate, putative capacity to accumulate soluble carbohydrates, and tillering capacity) that may influence the capacity of spring wheat to recover after defoliation. Defoliated and undefoliated crops were compared to assess physiological differences between cultivars including recovery of biomass, leaf area and radiation interception at anthesis, and subsequent crop grain yield and yield components. All genotypes had similar responses to defoliation treatments indicating that the physiological attributes studied played little part in mitigating yield penalties after defoliation. Despite some differences in yield components amongst cultivars, defoliation did not adversely affect cultivars with different yield component combinations under non-water limited conditions. Later and intense defoliation (around GS30/31) resulted in large yield penalties (40%) which reduced both grain number and kernel mass. However, earlier defoliation (before GS28) induced small or insignificant yield penalties. Defoliation often reduced canopy radiation interception and crop biomass at anthesis but this rarely translated into large yield penalties. These studies further demonstrate that shorter season spring cereals can provide valuable forage (up to 1.2 t DM/ha) for grazing during early vegetative growth without inducing large yield penalties. This study suggests that beyond appropriate phenology, there were no other specific characteristics of cultivars that improved the recovery after grazing. Hence farmers don’t need specific dual-purpose cultivars and can still focus on those that optimize grain yield potential for a particular environment and sowing date. The timing and intensity of defoliation appear to be larger drivers of yield recovery in spring cereals and better understanding of these relationships are needed to provide grazing management guidelines that mitigate risk of yield penalties in dual-purpose cereal crops.

## Introduction

Grazing cereal grain crops during their vegetative phase and then allowing the crop to recover to produce grain yield (dual-purpose crop) offers the potential to substantially increase productivity, profitability and flexibility on mixed crop-livestock farms (Dove and Kirkegaard, 2014). Dual-purpose crops have been used for many years and are widely adopted in south-eastern Australia (Harrison et al., 2011a) and in the Great Plains of the United States (Cutler et al., 1949; Carver et al., 2001). Traditionally these systems have involved grazing slower-developing winter cereals which have a significant vernalization requirement to generate a longer vegetative period for grazing, as well as an extended period for post-grazing recovery of crop biomass and grain yield. Consequently, the winter cultivars are developed to suit environments with early sowing opportunities and longer growing seasons with higher rainfall (Bell et al., 2015b) and breeding is focused on winter phenology, and resistance to disease resistance and weather damage (Carver et al., 2001; Hunt, 2017). In these regions, mixed farms obtain large benefits from the highly valuable forage for livestock production, and the additional revenue and income diversification provided by the grain production (Bell et al., 2015a). However, recently there has been increasing interest in the potential to obtain valuable grazing from high protein wheat crops on mixed farms in environments with shorter growing seasons, where faster developing spring wheats are better suited and more commonly grown. In these cases, the income from grain is the focus, however, economically valuable grazing potential has been demonstrated in both simulation (Moore, 2009; Bell et al., 2015b; Hussein et al., 2017) and experimental studies (McMullen and Virgona, 2009; Seymour et al., 2015; Sprague et al., 2018) from a range of different cultivars without yield penalties with careful grazing management.

Avoiding large grain yield penalties from the grazing is critical in order to maximize the value of dual-purpose crops. Recent reviews of historical studies on dual-purpose crops have shown that grazing reduces grain yield by about 7% (Edwards et al., 2011; Harrison et al., 2011a; Bell et al., 2014). Amongst previous studies large yield penalties can occur and are generally related to late and severe defoliation (or grazing), when either the reproductive structures (developing spikes) are directly removed, tiller number was significantly reduced and/or there was insufficient time for adequate crop recovery to achieve the required canopy cover to set the same yield potential (grain number) as the un-grazed crops (Harrison et al., 2011c; Butchee and Edwards, 2013). Grazing prior to the initiation of reproductive development [*equiv.* growth stage (GS) 31, stem elongation or jointing; Zadoks et al., 1974] is recommended to avoid grain yield reduction through direct removal of heads or tillers (Virgona et al., 2006). These general observations related to late grazing are consistent for winter cereal cultivars that have been used for dual-purpose. However, relatively little has been done to understand the dynamics of regrowth and physiological factors and processes involved in recovery after grazing in different genotypes (Harrison et al., 2011b,c). Some experiments have found larger trade-offs between grazing and grain yield for spring cultivars than in slower-developing winter cultivars (Sprague et al., 2018), while others have found similar responses in winter and spring types (Royo and Romagosa, 1996; Royo, 1997). Taller genotypes have been found to have less yield reduction from grazing than newer semi-dwarf cultivars, due to differences in susceptibility to lodging and yield potential (Winter and Thompson, 1990), but differences in growth habit amongst semi-dwarf genotypes (erect vs. prostrate) were not shown to differ in response to defoliation (Butchee and Edwards, 2013). This is likely to be related to interactions with environment and possibly physiological differences between cultivars in their yield setting attributes. In spring cereals, where both the grazing and recovery periods are short, there is a greater risk of yield penalties than for winter cereals in longer-season environments. The faster development through the vegetative period means they have less time to recover after defoliation and it is therefore more difficult to achieve critical radiation interception or biomass during the critical period determining grain number (Fischer, 1985). Hence, a better understanding of crop recovery and the genotypic attributes or management interventions that may mitigate risks of yield penalties after grazing are of interest.

Most research investigating the factors affecting recovery after defoliation has been conducted in slow-developing winter cereals, with far less understanding in faster-developing spring varieties (Harrison et al., 2011a). Some preliminary assessments of grain yield recovery among commonly grown spring wheat cultivars have been conducted in a range of environments across western (Seymour et al., 2015) and southern Australia (Frischke et al., 2015; Latta, 2015). There were variable amounts of recovery after defoliation, which were not clearly related to seasonal conditions nor to specific cultivars. In addition, many of the studies implemented defoliation treatments (mowing or grazing) across all cultivars at the same time, so that the timing of defoliation in relation to the phenological growth stage of the crop was not consistent across all genotypes. Confounding defoliation timing with crop development stage makes it difficult to determine whether varietal characteristics other than phenology were related to the outcome. Further, these defoliation treatments also often interact with water supply to the crop which can induce different responses after grazing (Virgona et al., 2006).

Given the lack of knowledge and inconsistent results in previous studies where recovery after defoliation in spring cereals has been investigated, we aimed to investigate cultivar differences based on putative physiological or morphological attributes thought to infer a greater capacity to recover and re-establish yield potential after grazing. Firstly, yield penalties are often associated with a reduction in tiller number as a driver of grain number, so that cultivars with a greater tillering capacity may provide greater resilience to defoliation. Secondly, crop phenology may also influence recovery independently of the interaction with grazing time (i.e., pre-GS31). Finally, some cultivars have a greater tendency to accumulate soluble carbohydrates (CHO) in the stem prior to anthesis and rely on these resources to fill the grain (van Herwaarden et al., 1998). It is likely CHO accumulation will be reduced by vegetative defoliation (Tian et al., 2012; Hu et al., 2019), so that cultivars with a greater reliance on stem CHO may incur greater yield penalties. Here we report on a series of experiments which hypothesized that we would expect physiological differences between cultivars to bring about either improved or reduced capacity for yield recovery after defoliation. Such characteristics may provide breeding targets, or varietal selection guides for those wishing to utilize spring cereals as a dual-purpose crop. Our experiments were implemented in a way that mitigated the interactions of environment (water and nitrogen supply), and hence aimed to draw out the genotype by management interactions in the absence of the confounding influences of water or nutrient stresses.

## Materials and Methods

### Experimental Approach

Four experiments were conducted between 2011 and 2013 in southern Queensland, Australia to examine the effect of defoliation on regrowth and grain yield recovery of different cultivars of spring wheat and barley. Cultivars of wheat and barley were chosen to evaluate whether specific traits or characteristics influenced grain yield recovery after defoliation. Traits compared amongst genotypes included the rate of phenological development (e.g., faster- vs. slower-developing types), tillering capacity and accumulation of water-soluble carbohydrates in stems prior to grain fill (Table 1). All cultivars chosen were commercially relevant at the time of the study and grown by farmers across the region. Defoliation was implemented using a self-propelled sickle-bar mower at approximately 3 cm height to simulate an intense grazing event. Timing of defoliation was tailored to match phenological stages in each cultivar (i.e., defoliation may occur on different days). Thus, the confounding effect of cultivars being defoliated at different development stages was minimized, as this is already known to have a significant influence on grain yield recovery. In some cases, sowing date was altered to try to achieve synchronous flowering between cultivars to remove another significant confounding factor on yield development. All crops were grown using agronomic recommendations for grain-only production systems (e.g., sowing densities 100–170 plants/m^2^, sowing times) and were exposed to only a single defoliation event corresponding with the rationale that these short-season spring wheats would be grown for grain production and provide only opportunistic grazing for a short period (2–3 weeks).

**TABLE 1:** Experimental design, locations, and cultivars used to compare phenology (fast *vs*. slow), soluble carbohydrate accumulation (CHO) and tillering capacity related to response after defoliation in spring wheats.

Exp. #	Design	Location	Latitude, Longitude	Year	Water regime	*Varietal attributes*		
						***Phenology***		
1	Phenology × Defoliation timing	Gatton, QLD	27°32.5′S, 152°20.3′E	2011	Irrigated	Slow	*cv. Gregory*	
						Fast	*cv. Crusader*	
	
2	Phenology × CHO accumulation	Norwin, QLD	27°33.0′S, 151°19.6′E	2012	Rainfed		**High CHO**	**Mod. CHO**
	
						Slow	*cv. Gregory*	*cv. Yenda*
						Fast	*cv. H45*	*cv. Crusader*
	
3	Phenology × CHO accumulation	Brookstead, QLD	27°43.4′S, 151°17.5′E	2013	Irrigated		**High CHO**	**Mod. CHO**
	
						Slow	*cv. Gregory*	*cv. Yenda*
						Fast	*cv. H45*	*cv. Crusader*
	
4	Phenology × Tillering capacity	Brookstead, QLD	27°43.4′S, 151°17.5′E	2013	Irrigated		**Low tillering**	**High tillering**
	
						Fast	*cv. Gregory*	*cv. Sunvale*
						Slow	*cv. Gladius*	*cv. Bolac*
						Barley	*cv. Scope*	*cv. Hindmarsh*

All experiments were conducted on black vertosol soils (Isbell, 1996) which are widely used for grain production in this region of Australia. These are moderate-heavy clay soils with high water-holding capacity (200–260 mm plant-available water-holding capacity), high fertility (Colwell *P* > 50 mg/kg, organic matter > 3%) and neutral pH (7–8). Three of the experiments were conducted under irrigation to remove any confounding influences of water stress in response of different genotypes. One experiment (Experiment 2) was rainfed but was sown on a full soil profile and crops did not experience moisture stress that would significantly reduce yield potential. All experiments were managed to ensure N was not limiting by applications of urea at sowing and throughout the growing season prior to irrigations. All experiments were maintained weed free through the application of broadleaf selective herbicides approximately 6 weeks after sowing, hand weeding as necessary, and preventative insect and rust sprays were applied during the season. The cultivar Gregory was used across all experiments to allow for inter-comparisons between them.

### Experimental Implementation and Details

#### Experiment 1. Phenology × Defoliation Stage (Gatton, 2011)

The field experiment was conducted at Gatton Research Station, Queensland between May and November 2011, under fully irrigated conditions. Four replicates (2 × 10 m) in a randomized complete block design of four defoliation treatments GS25, GS28, and GS31 (dates provided in Table 2) and an uncut control were implemented on two cultivars. The cultivars Gregory (slower-developing) and Crusader (faster-developing) were sown in 25 cm rows on 16 May and 3 June 2011, respectively, aiming to achieve synchronous flowering time (7 September 2011), based on thermal time differences between cultivars. Hence, the different defoliation timings occurred at 50, 57, and 67 days after sowing for Gregory and 39, 49, and 55 days after sowing for Crusader. A plant population of 170 plants/m^2^ was established in both cultivars. The experiment was provided with regular weekly irrigation (overhead sprinklers) to balance potential evapotranspiration to ensure the crops were not water stressed during recovery.

**TABLE 2:** Biomass removed and subsequent leaf area index (LAI), radiation interception (R_*i*_) and tillers at anthesis (#/m^2^) for two spring wheat cultivars with different phenological development rate (mid-season; *cv*. Gregory – Greg. and: *cv*. Crusader – Crus.) following defoliation at different growth stages compared to an undefoliated control (Experiment 1 – Gatton, 2011).

	At defoliation				At anthesis									
Defoliation timing	Date^*A*^		Removed biomass (t/ha)		LAI		R_*i*_		All tillers (#/m^2^)		Main tillers (#/m^2^)		Minor tillers (#/m^2^)	
	Crus.	Greg.	Crus.	Greg.	Crus.	Greg.	Crus.	Greg.	Crus.	Greg.	Crus.	Greg.	Crus.	Greg.
Uncut					6.37	6.00	0.97	0.96	1110	1380	770	890	340	470
GS 25	12 July	5 July	0.55	1.16	4.67	4.02	0.93	0.90	1330	1840	1030	920	310	920
GS 28	22 July	12 July	0.78	1.73	3.45	3.17	0.94	0.82	1230	1680	940	870	290	800
GS 31	28 July	22 July	2.03	2.28	2.33	2.70	0.74	0.75	990	1380	550	640	460	720

			***P***	***LSD***	***P***	***LSD***	***P***	***LSD***	***P***	***LSD***	***P***	***LSD***	***P***	***LSD***

*Genotype*			<0.001	0.25	0.323		0.623		<0.001	168	0.641		<0.001	118
*Defoliation*			<0.001	0.31	<0.001	0.67	<0.001	0.067	0.005	235	<0.001	168	0.084	168
*Genotype × Defoliation*			0.124		0.464		0.925		0.740		0.92		0.029	252

**P* score (P) for main effects of genotype, defoliation treatment and the interaction are provided below and least significant difference (LSD at *P* = 0.05) for these effects when they are significant. ^*A*^Defoliation was timed to achieve the same growth stage for the two cultivars, sown on different dates.*

#### Experiment 2. Phenology and CHO Accumulation (Norwin, 2012)

The experiment was conducted in a farm field at Norwin, Queensland between May and November 2012 under rainfed conditions. The experiment included cultivars paired for phenology type (slower and faster developing cultivars) which are known to have a higher or lower tendency to accumulate water soluble carbohydrates (CHO) that can be translocated during grain filling (Ruuska et al., 2006; Neil Fettell, personal communication). Water-soluble carbohydrate accumulation was not measured here. The experimental design was a split-plot design with three replicated blocks with cultivars as main plots and defoliation treatments sub-plots (15 × 2 m). The slower developing cultivars (Gregory and Yenda) were sown on 17 May, and faster developing cultivars (H45 and Crusader) were sown on 23 June. Lack of sowing rain at the appropriate time meant this difference in sowing date was larger than anticipated to achieve synchronous flowering. All cultivars were sown in 25 cm rows and established 100–120 plants/m^2^. Cultivars were defoliated at GS30/31 at 54 days after sowing, on 10 July for the slower developing cultivars and 16 August for the faster developing cultivars. The earlier sown cultivars reached anthesis on 14 September and the later sown cultivars on 28 September. The site had been managed as a long fallow (18 months) prior to the experiment and had a full soil profile (250 mm plant available water) with high levels of soil nitrogen (>400 kg NO_3_/ha) at sowing.

#### Experiment 3. Phenology and CHO Accumulation (Brookstead, 2013)

This experiment was located on a farm at Condamine Plains, near Brookstead on the eastern Darling Downs, Queensland in 2013. It repeated the treatments in Experiment 2 but implemented them under irrigation. A fully randomized block design with three replicates and 8 plots (2 × 12 m) included a defoliated and undefoliated treatment for 4 cultivars. All genotypes were sown on the same date (18 June 2013), with a row spacing of 40 cm and an established plant density of 90–110 plants/m^2^. There was little difference in early phenological development of cultivars, so all genotypes were defoliated on 6 August (49 days after sowing) when still vegetative and at GS 26 (Zadoks et al., 1974). Irrigation was applied to ensure no water limitations with a total of 40 mm of water supplied per week (balanced for any rainfall). Water soluble carbohydrates were not measured here.

#### Experiment 4. Phenology and Tillering Capacity (Brookstead, 2013)

This field experiment was established at Condamine Plains, near Brookstead on the eastern Darling Downs, Queensland in 2013 (at the same farm as Experiment 3). In this experiment four spring wheat and two spring barley cultivars were chosen to represent a range of varying tillering capacity (i.e., the number of ears produced per m^2^ at maturity) (see Table 1). The same experimental design (three replicates in randomized block design) and crop management was implemented as in Experiment 3. The tiller dynamics of defoliated and undefoliated crops were monitored up until anthesis to determine any differences in tiller development between cultivars under defoliation (details outlined below).

### Crop Measurements

#### Crop Biomass, Yield and Yield Components

In all experiments, biomass removed during defoliation from mowing at approximately 3 cm height and residual biomass was measured by taking quadrat cuts (0.6 – 1.0 m^2^) before and after defoliation. At maturity, larger quadrat cuts (1.5 – 2.0 m^2^) were taken to ground level from the center of each plot to determine grain yield and maturity biomass; any senesced leaf material was also collected. These samples were dried for 3 days at 80°C before being weighed. The number of ears in each sample was counted to determine ear number per m^2^ and these were subsequently threshed and cleaned. Grain samples were then dried at 80°C and weighed and a subsample of 100 grains from each sample was taken and weighed to determine average kernel mass. Calculations of other yield components were then based on these measured attributes; harvest index (grain yield/maturity biomass), grain number per m^2^, kernels per ear.

#### Tiller Dynamics

In Experiments 1 and 4, the number of primary, secondary and senesced tillers were recorded between the initiation of reproductive development and anthesis. After defoliation was implemented the number of tillers emerging was monitored on a set of 7–10 marked plants in each plot. At booting (GS45) and/or start of anthesis (GS60) a destructive sample (quadrat 0.5–1.0 m^2^) was taken to determine the number of tillers present at these times. Primary tillers were identified as those that had produced a flag leaf or started anthesis at these respective times, while secondary tillers had not reached these development stages yet. The ratio of the number of tillers compared to the final ear numbers gave some indication of the proportion of tillers that had senesced during grain filling.

#### Crop Leaf Area Index and Radiation Interception

Crop radiation interception and predicted leaf area index (measured with a Decagon’s AccuPAR model LP-80 PAR/LAI Ceptometer) was measured at anthesis (GS 65) in all experiments. Two measures above canopy height were matched with four measures at ground level below the canopy spanning 3 or 4 plant rows in each plot. Leaf distribution value (*X*) was set to 0.96 as recommended for wheat (Decagon Instruction Manual).

### Statistical Analysis

Statistical analysis used two-way analysis of variance in GenStat version 19.1 (VSN International Ltd), with the main effects of genotype and defoliation treatments. Interactions between these two factors were expected if they responded differently to defoliation treatments. Fischers’ protected least significant difference (LSD) was used for mean separation where appropriate. Experiment 4 was also analyzed for species and excluding the two barley genotypes to see if any differences amongst the four wheat genotypes were evident. As there were no genotype by defoliation interactions across experiments, using data from all genotypes and experiments, causal relationships of defoliation effects on plant growth (post-defoliation growth, anthesis biomass and canopy interception, post-anthesis growth), yield and yield components (grain yield, maturity biomass, harvest index, grain number per m^2^, kernel mass, kernels per ear, ears per m^2^) were explored through multiple regressions. To allow for comparisons across experiments where the magnitude of effects differed, we calculated the relative value of the defoliated crop as a proportion of the undefoliated crop. Where there was likely causation and significant correlations (*P* < 0.05) the line-of-best-fit between the predictor and response variable was derived using Microsoft Excel using least squares regression.

## Results

### Experiment 1. Phenology × Defoliation Timing (Gatton, 2011)

Delaying defoliation timing until later phenological stages allowed significant increases in biomass removed in both cultivars. Gregory had more biomass than Crusader at each of the defoliation timings as this occurred 8–12 days after sowing later than in Crusader (Table 2). The defoliation treatments significantly reduced LAI at anthesis compared to the undefoliated control in both cultivars, and later defoliation had lower LAI. Radiation interception (Ri) at anthesis was also significantly reduced in both cultivars after the latest defoliation timing, but there was no significant difference in earlier defoliation times in Crusader, or the earliest timing in Gregory.

At anthesis the number of tillers per plant was increased by earlier defoliation (GS25/28) compared to the undefoliated control. This was particularly evident in the higher tillering cultivar Gregory due to an increase in the number of secondary tillers. In both cultivars, later defoliation (GS31) had a similar number of tillers at anthesis to the undefoliated treatments but there was a reduction in primary tillers and more secondary tillers in this treatment.

Both cultivars had similar grain yields and maturity biomass across the defoliation treatments and there was no significant interaction between cultivar and defoliation treatments (Table 3). There was a 40% reduction in both crop biomass and grain yield following defoliation at GS31. The earliest defoliation treatment did not reduce grain yield significantly (<10%), but the defoliation at GS28 reduced grain yield and crop biomass. Reductions in grain number were the main driver of the yield reductions particularly in the latest defoliation, with kernel mass also significantly reduced in the latest defoliation time in both cultivars. The two cultivars responded differently to defoliation in terms of grain number reduction, as shown by the significant interactions for both ears/m^2^ and kernels/ear. Gregory maintained ear number in all but the earliest defoliation timing (GS25), where ear number was reduced by 24%. This was compensated through an increase in kernels/ear (20%) but grain number was still reduced. The reason for this reduction is unclear. In Gregory the later defoliation did not reduce ear number but did reduce kernels per ear and hence reduced grain number. In Crusader, late defoliation (GS31) reduced ear number more than for Gregory, but the kernels per ear were less affected.

**TABLE 3:** Grain yield and yield components at harvest of two spring wheat cultivars with different phenological development rate [slower: *cv*. Gregory (Greg.) and faster: *cv*. Crusader (Crus.)] following defoliation at different growth stages compared to an undefoliated control (Experiment 1 – Gatton, 2011).

Defoliation timing	Grain yield (t/ha)		Biomass (t/ha)		Harvest index		Grain no. ‘000/m^2^		Ears/m^2^		Kernels/ear		Kernel mass (mg)	
	Crus.	Greg.	Crus.	Greg.	Crus.	Greg.	Crus.	Greg.	Crus.	Greg.	Crus.	Greg.	Crus.	Greg.
Uncut	6.24	6.00	14.5	15.1	0.43	0.40	19.4	14.9	490	444	39.9	34.2	32.2	40.4
GS 25	5.88	5.35	13.5	12.1	0.43	0.44	17.9	13.7	492	339	36.7	40.7	32.9	39.2
GS 28	5.13	5.40	11.3	12.0	0.45	0.45	15.7	14.3	431	424	36.6	33.8	32.6	37.7
GS 31	3.53	3.71	8.1	8.9	0.44	0.42	11.9	10.7	395	449	30.3	23.6	29.8	35.0

	***P***	***LSD***	***P***	***LSD***	***P***	***LSD***	***P***	***LSD***	***P***	***LSD***	***P***	***LSD***	***P***	***LSD***

*Genotype*	0.728	–	0.667	–	0.042	0.01	<0.001	1.5	0.125	–	0.042	2.7	<0.001	1.1
*Defoliation*	<0.001	0.67	<0.001	1.4	0.001	0.02	<0.001	2.1	0.440	–	<0.001	3.8	<0.001	1.5
*Gen. × Defol.*	0.574	–	0.314	–	0.086	–	0.233	–	0.040	99	0.033	5.4	0.166	–

**P-*score (*P*) for main effects of genotype, defoliation treatment and the interaction are provided below and least significant difference (LSD at *P* = 0.05) for these effects when they are significant.*

### Experiment 2. Phenology and CHO Accumulation (Norwin, 2012)

All cultivars had similar biomass removal (0.94–1.25 t DM/ha) when defoliated at GS 30 (54 days after sowing), even though this occurred on different dates in the different phenology types (Table 4). Defoliation reduced crop biomass, LAI and radiation interception at anthesis significantly in all cultivars. Faster developing cultivars produced less biomass and leaf area by anthesis than the earlier sown slower developing cultivars, but there was no significant interaction with defoliation for anthesis biomass and LAI. There was very low radiation interception and leaf area in the defoliated later-sown fast-developing genotypes and the penalty was significantly larger than in the slower developing genotypes. There was large variance in the onset of flowering amongst tillers in these fast-developing genotypes and further leaf area accumulation occurred after our sampling allowing the crop to compensate further after defoliation.

**TABLE 4:** Biomass removed and subsequent biomass, leaf area index (LAI), and radiation interception (*R*_*i*_) at anthesis of four spring wheat cultivars following defoliation at GS30/31 (DEF) compared to an undefoliated control (UN) (Experiment 2 – Norwin, 2012).

			At defoliation		At anthesis						
Phen.	CHO accum.	Cultivar	Date	Biomass removed (t/ha)	Date	Biomass (t/ha)		LAI		Ri	
					
						UN	DEF	UN	DEF	UN	DEF
Slow	High	Gregory	10 July	1.22	14 September	10.90	9.18	6.18	5.05	0.94	0.88
	Low	Yenda	10 July	1.25	14 September	10.00	8.07	5.83	4.55	0.91	0.84
Fast	High	H45	16 August	0.94	28 September	8.24	5.29	3.46	1.96	0.74	0.55
	Low	Crusader	16 August	1.25	28 September	8.91	5.69	3.34	2.05	0.71	0.56

				*P*		*P*	*LSD*	*P*	*LSD*	*P*	*LSD*

*Genotype*				0.541		<0.001	0.53	<0.001	0.35	<0.001	0.04
*Defoliation*						<0.001	0.38	<0.001	0.25	<0.001	0.03
*Genotype x Defoliation*						0.127		0.717	–	0.003	0.05

*Cultivars differ in phenological development rate (slower: *cv*. Gregory, Yenda, and faster: *cv*. Crusader, H45) and capacity to accumulate water soluble carbohydrates (CHO) in biomass before flowering. *P* score (*P*) for main effects of genotype, defoliation treatment and the interaction are provided below and least significant difference (LSD at *P* = 0.05) for these effects when they are significant.*

Despite the significant reductions in anthesis biomass and radiation interception, by crop maturity there were no significant effects of defoliation on crop grain yield, biomass or yield components (Table 5). Grain yield and biomass varied <8% across all genotypes, and similar levels of variation occurred for the various yield components. While there were genotype differences in crop yield, biomass and yield components demonstrating different combinations of yield components amongst the cultivars, there was no interaction between defoliation and genotype for any of these attributes (Table 5).

**TABLE 5:** Grain yield and yield components at harvest of four spring wheat cultivars following defoliation at GS30 (DEF) compared to an undefoliated control (UN) (Experiment 2 – Norwin, 2012).

Phen.	CHO accum.	Cultivar	Grain yield (t/ha)		Biomass (t/ha)		Harvest index		Grain no. (‘000/m^2^)		Ears/m^2^		Kernel mass (mg)		Kernels/ear	
			UN	DEF	UN	DEF	UN	DEF	UN	DEF	UN	DEF	UN	DEF	UN	DEF
Slow	High	Gregory	4.52	4.16	14.2	13.0	0.32	0.32	14.5	13.5	463	434	31.1	30.8	31.4	31.1
	Low	Yenda	3.74	3.54	12.7	12.0	0.29	0.29	15.9	15.6	428	463	23.3	22.5	36.8	34.0
Fast	High	H45	5.10	5.27	11.1	12.2	0.46	0.44	17.8	18.3	426	476	28.7	28.7	41.7	38.2
	Low	Crusader	4.71	4.55	11.1	11.7	0.42	0.39	18.7	17.4	534	520	25.5	26.0	34.9	33.1

			***P***	***LSD***	***P***	***LSD***	***P***	***LSD***	***P***	***LSD***	***P***	***LSD***	***P***	***LSD***	***P***	***LSD***

*Genotype*			<0.001	0.70	0.027	1.5	<0.001	0.03	0.003	2.4	0.001	44	<0.001	1.6	<0.001	3.2
*Defoliation*			0.584		0.952		0.101		0.525		0.516		0.793		0.067	
*Genotype × Defoliation*			0.890		0.429		0.415		0.868		0.220		0.844		0.737	

*Cultivars differ in phenological development rate (slower: *cv*. Gregory, Yenda, and faster: *cv*. Crusader, H45) and capacity to accumulate water soluble carbohydrates (CHO) in biomass before flowering. *P-*score (*P*) for main effects of genotype, defoliation treatment and the interaction are provided below and least significant difference (LSD at *P* = 0.05) for these effects when they are significant.*

### Experiment 3: Phenology and CHO Accumulation (Brookstead, 2013)

All cultivars produced similar biomass at the time of defoliation (0.4 – 0.5 t/ha), but this was less than observed in the similar experiment the previous year due to an earlier defoliation timing (GS26) and a later sowing date. Despite this smaller biomass removal, there was a significant reduction in anthesis DM in all cultivars (0.9–2.0 t/ha), but this was not reflected in radiation interception at anthesis (Table 6). The faster-developing cultivars (H45 and Crusader) had lower LAI and radiation interception than the slower-developing cultivars at this time.

**TABLE 6:** Biomass removed and subsequent biomass, leaf area index (LAI), and radiation interception (R_*i*_) at anthesis of four spring wheat cultivars following defoliation at GS26 (DEF) compared to an undefoliated control (UN) (Experiment 3 – Brookstead, 2013).

Cultivar	Removed biomass (t/ha)	Anthesis DM (t/ha)		Anthesis LAI		Anthesis Ri	
		UN	DEF	UN	DEF	UN	DEF
Gregory	0.37	8.51	7.62	6.58	6.44	0.94	0.94
Yenda	0.48	7.61	6.51	7.03	6.41	0.96	0.94
H45	0.40	8.08	6.07	4.79	4.56	0.88	0.88
Crusader	0.42	8.56	7.68	4.57	3.82	0.86	0.82

	***P***	***P***	***LSD***	***P***	***LSD***	***P***	***LSD***

*Genotype*	0.541	0.019	0.84	<0.001	0.93	<0.001	0.05
*Defoliation*	na	<0.001	0.59	0.176	–	0.286	–
*Gen. x Def.*	na	0.449		0.874	–	0.701	–

*Cultivars differ in phenological development rate (slower: *cv*. Gregory, Yenda, and faster: *cv*. Crusader, H45) and capacity to accumulate water soluble carbohydrates (WSC) in biomass before flowering. *P-*score (*P*) for main effects of genotype, defoliation treatment and the interaction are provided below and least significant difference (LSD at *P* = 0.05) for these effects when they are significant.*

As observed in the previous similar experiment 2, there was no significant effect of defoliation on grain yield or maturity biomass across the cultivars (Table 7). Grain yield and maturity biomass varied <8% across all genotypes except for Yenda. Yenda had greater differences between defoliated and undefoliated treatments in grain yield, biomass and particularly in grain number per m^2^, but these were not statistically significant. There was a significant reduction in kernel mass due to defoliation, but all other grain yield components were unaffected. Again, there were clear genotypic differences in crop yield, biomass and yield components demonstrating different combinations of yield components amongst cultivars, but there was no interaction between defoliation and genotype for any of these attributes (Table 7).

**TABLE 7:** Grain yield and yield components at harvest of four spring wheat cultivars following defoliation at GS26 (DEF) compared to an undefoliated control (UN) (Experiment 3 – Brookstead, 2013).

Cultivar	Grain yield (t/ha)		Biomass (t/ha)		Harvest index		Grain no. (‘000/m^2^)		Ears/m^2^		Kernel mass (mg)		Kernel no./ear	
	UN	DEF	UN	DEF	UN	DEF	UN	DEF	UN	DEF	UN	DEF	UN	DEF
Gregory	4.33	4.58	12.7	12.5	0.34	0.37	14.1	15.7	476	479	30.8	29.3	29.6	32.8
Yenda	4.46	3.85	13.6	12.1	0.33	0.32	19.0	17.7	543	514	23.6	21.7	34.9	34.6
H45	5.56	5.10	12.1	11.1	0.46	0.46	17.3	17.5	421	408	32.1	29.4	41.2	43.0
Crusader	4.82	4.44	11.0	10.6	0.44	0.41	17.1	16.4	446	462	28.3	26.7	38.1	35.6

	***P***	***LSD***	***P***	***LSD***	***P***	***LSD***	***P***	***LSD***	***P***	***LSD***	***P***	***LSD***	***P***	***LSD***

*Genotype*	0.029	0.75	0.046	1.55	<0.001	0.03	0.125		0.010	62	<0.001	3.2	<0.001	3.6
*Defoliation*	0.220		0.161		0.661		0.963		0.778		0.084	2.2	0.684	
*Gen. × Def.*	0.614		0.769		0.230		0.736		0.861		0.974		0.388	

*Cultivars differ in phenological development rate (slower: *cv*. Gregory, Yenda, and faster: *cv*. Crusader, H45) and capacity to accumulate water soluble carbohydrates (WSC) in biomass before flowering. *P-*score (*P*) for main effects of genotype, defoliation treatment and the interaction are provided below and least significant difference (LSD at *P* = 0.05) for these effects when they are significant.*

### Experiment 4: Phenology and Tillering Capacity (Brookstead, 2013)

At defoliation (GS26), the barley cultivar Scope has significantly more biomass than the all other wheat and barley genotypes (Table 8). Generally, the higher tillering cultivars had a less erect habit which reduced the amount of biomass removed by mowing, but this was not statistically significant.

**TABLE 8:** Biomass removed, tillers per m^2^ (major and minor) present at booting and subsequent biomass, leaf area index (LAI), and radiation interception (*R*_*i*_) at anthesis of four spring wheat cultivars following defoliation at GS26 (DEF) compared to an undefoliated control (UN) (Experiment 4 – Brookstead, 2013).

Crop	Tillering	Cultivar	Removed DM (t/ha)	Anthesis						Booting					
				Biomass (t/ha)		LAI		Ri		All tillers (#/m^2^)		Major tillers (#m^2^)		Minor tillers (#/m^2^)	
				UN	DEF	UN	DEF	UN	DEF	UN	DEF	UN	DEF	UN	DEF
Wheat	Low	Gregory	0.43	8.30	6.45	5.79	5.44	0.93	0.92	705	563	378	302	327	261
		Gladius	0.44	7.69	6.64	3.59	3.54	0.76	0.76	566	562	293	302	273	260
	High	Sunvale	0.35	7.74	6.12	6.41	5.34	0.95	0.89	1053	1108	466	557	587	551
		Bolac	0.31	7.47	5.68	5.40	4.05	0.90	0.83	436	460	221	248	215	212
Barley	Low	Scope	0.95	8.05	6.15	6.73	6.20	0.95	0.95	888	1212	382	643	505	569
	High	Hindmarsh	0.52	7.72	7.47	5.95	5.14	0.94	0.88	878	1467	364	822	513	644

			**LSD**	***P***	***LSD***	***P***	***LSD***	***P***	***LSD***	***P***	***LSD***	***P***	***LSD***	***P***	***LSD***

*Genotype*			*0.28*	0.232	–	<0.001	0.56	<0.001	0.03	<0.001	220	<0.001	96	<0.001	163
*Defoliation*				<0.001	0.49	<0.001	0.32	<0.001	0.02	0.074	–	<0.001	58	0.960	
*Genotype × Defoliation*				0.315	–	0.211	–	0.038	0.04	0.298	–	0.005	134	0.941	

*Cultivars differ in tillering capacity but have a similar phenological development rate. *P-*score (P) for main effects of genotype, defoliation treatment and the interaction are provided below and least significant difference (LSD at *P* = 0.05) for these effects when they are significant.*

Defoliation had no effect on the total number of tillers produced at booting (Table 8). The number of primary tillers was significantly reduced in the higher tillering cultivars (Sunvale, Scope, and Hindmarsh) and compensated by more secondary tillers, but no differences were observed in the lower tillering cultivars. The number of tillers at booting (GS39) did not necessarily correspond to the expected classifications across the various genotypes, but the final ear numbers per m^2^ (Table 9) did, indicating that the higher tillering varieties produce more secondary tillers that result in grain producing ears. The two barley cultivars and wheat cv. Sunvale had significantly more tillers (both primary and secondary tillers) than the other wheat genotypes.

**TABLE 9:** Grain yield and yield components at harvest of four spring wheat and two spring barley cultivars following defoliation at GS26 (DEF) compared to an undefoliated control (UN) (Experiment 4 – Brookstead, 2013).

Crop	Tillering	Cultivar	Grain yield (t/ha)		Biomass (t/ha)		Harvest index		Grain no. ‘000/m^2^		Ear no./m^2^		Kernel no./ear		Kernel mass (mg)	
			UN	DEF	UN	DEF	UN	DEF	UN	DEF	UN	DEF	UN	DEF	UN	DEF
Wheat	Low	Gregory	5.01	4.54	13.4	12.0	0.37	0.38	16.7	16.0	458	455	36.5	35.2	30	28
		Gladius	4.88	4.16	12.0	10.4	0.41	0.40	14.8	14.1	446	477	33.3	29.5	33	29
	High	Sunvale	4.91	4.73	12.4	12.0	0.40	0.39	19.3	19.0	618	657	31.3	28.8	25	25
		Bolac	4.73	4.04	13.3	11.4	0.35	0.35	18.5	17.9	527	520	35.3	34.4	26	23
Barley	Low	Scope	3.47	3.48	12.1	9.5	0.29	0.37	8.5	9.2	652	640	13.1	14.4	41	38
	High	Hindmarsh	4.75	4.71	11.9	10.0	0.40	0.47	11.9	13.3	774	758	15.4	17.7	40	35

			***P***	***LSD***	***P***	***LSD***	***P***	***LSD***	***P***	***LSD***	***P***	***LSD***	***P***	***LSD***	***P***	***LSD***

*Genotype*			<0.001	0.50	0.001	0.99	<0.001	0.025	<0.001	1.2	<0.001	80	<0.001	2.25	<0.001	2.4
*Defoliation*			0.016	0.29	<0.001	0.57	0.002	0.014	0.752	–	0.824	–	0.185	–	<0.001	1.4
*Genotype × Defoliation*			0.552	–	0.25	–	0.002	0.035	0.387	–	0.964	–	0.104	–	0.583	–

*Cultivars differ in tillering capacity but have a similar phenological development rate. *P*-score (*P*) for main effects of genotype, defoliation treatment and the interaction are provided below and least significant difference (LSD at *P* = 0.05) for these effects when they are significant.*

Defoliation significantly reduced anthesis biomass, LAI and radiation interception at anthesis (Table 8). All genotypes had similar anthesis biomass, but defoliation reduced this by 1.0-1.9 t/ha across all genotypes except Hindmarsh barley. There were genotype differences in LAI and radiation interception and a significant interaction between genotype and defolation for radiation interception. This interaction showed that the higher-tillering genotypes (wheat and barley) had a greater reduction in radiation interception than the lower-tillering group.

Defoliation significantly reduced grain yield and maturity biomass across all genotypes, however, the reductions in grain yield were small (Table 9, 0.36 t/ha on average, ranging from 0.04 to 0.72 t/ha). Scope barley had lower yield than the other genotypes which all achieved a similar yield. There was no genotype by defoliation interaction in grain yield and maturity biomass, but there was a significant effect on harvest index. This interaction was because defoliation increased harvest index significantly (*P* < 0.01) in the barley genotypes but not in the wheat genotypes.

Of the yield components, defoliation reduced kernel mass significantly but there was no significant effect of defoliation on yield components related to grain number (i.e., ear number per m^2^ and kernels per ear). Among the genotypes, there were clear differences in yield components. As expected, the barley genotypes had less kernels per tiller and lower grain number per m^2^, but larger kernels. The wheat cultivars Sunvale and Bolac had significantly higher grain number but smaller kernels (24–25 mg) than Gregory and Gladius (29–31 mg). Cultivars Sunvale and Gladius had lower kernels per ear (30–31/ear) than Bolac and Gregory (35–36/ear), but these differences were compensated by differences in ear number per m^2^. Despite these apparent differences in tillering and yield components there was no significant interactions between genotype and defoliation (Table 9).

Further exploration of this data to examine if there were any significant effects related to tillering capacity (by grouping genotypes with similar tiller numbers) found no interactions, though there were differences between groups in ear number and kernels per ear, as indicated above. As the barley genotypes (particularly Scope) provided the main differences in the statistical analysis, a further analysis was conducted omitting the barley genotypes. However, this did not reveal any further statistical differences amongst wheat genotypes.

### Cross-Experiment Analysis of Defoliation Effects

**FIGURE 1:**
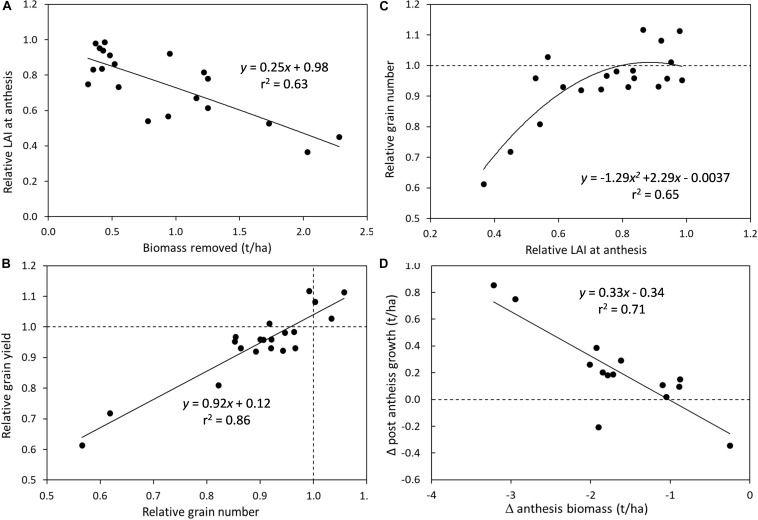
Crop defoliation effects on determinants of grain yield across experiments in spring cereals under water- and nitrogen-unlimited growing conditions: **(A)** Crop biomass removal effects on the relative leaf area index (LAI) at anthesis (defoliated/undefoliated); **(B)** Relationship between relative grain number and relative grain yield (defoliated/undefoliated); **(C)** Relative LAI at anthesis impacts on relative grain number (defoliated/undefoliated); and **(D)** change in anthesis biomass impacts on the change in post-anthesis growth.

Combining results across experiments demonstrate some of the critical drivers of grain yield reduction as a result of defoliation in spring cereal genotypes (Figure 1). There was a negative relationship between the amount of biomass removed by defoliation and the leaf area index (LAI) that was subsequently recovered by anthesis (Figure 1A). Every 1 t/ha of biomass removed resulted in a 25% reduction in LAI at anthesis. However, reductions of >40% in LAI at anthesis were required to dramatically reduce grain number, while lesser reductions in LAI had only small impacts on grain number (<10% decrease) (Figure 1C). The relative grain number (i.e., the ratio of grain number in defoliated vs. undefoliated crops) was closely correlated to relative grain yield, demonstrating that these reductions in grain number or sink limitations are the primary cause of yield penalties in defoliated crops (Figure 1B). The capacity for crops to compensate for the lower LAI and biomass at anthesis to achieve similar grain yields was shown by an increase in post-anthesis growth as the deficit in anthesis biomass increased (Figure 1D). However, this additional production was only 0.33 kg/kg of anthesis deficit so was not enough to fully recover maturity biomass.

## Discussion

We hypothesized that amongst spring cultivars with similar phenology, differences in physiological traits that influence how they establish grain yield would see them respond differently during recovery after defoliation. However, the experiments revealed no such evidence of genotypic differences in crop recovery after defoliation. There was no genotype by defoliation interactions on grain yield and few interactions in yield components between cultivars under defoliation compared to undefoliated crops. This result contrasts with studies on winter wheats that have shown differences in cultivar responses to defoliation (Thapa et al., 2010). Despite this lack of interaction between genotypic traits and defoliation, there was significant effects of defoliation timing on yield recovery. This indicates that genotype has less to do with the ability of the crop to recover after grazing than how the grazing is managed. It is clearly critical to manage the grazing to avoid later and more severe grazing to allow recovery of enough biomass and resources to maintain grain number and fill grains effectively. The research also clearly shows that spring wheat and barley genotypes could be used as a valuable forage source with little or no yield penalty associated with forage removal up to 1.2 t/ha and before GS31, even in environments which drive rapid crop development and with minimal terminal drought. However, the amount of biomass available for grazing is significantly less than that available from winter genotypes sown earlier (Dove and Kirkegaard, 2014; Hunt, 2017; Sprague et al., 2018).

### Genotype Effects on Grain Yield Response After Defoliation

The cultivars tested varied in three main attributes (phenological development rate, water soluble carbohydrate accumulation, and tillering capacity) which were thought to interact with defoliation, to either mitigate or intensify the effects on subsequent grain yield. However, across all experiments defoliation didn’t induce differential grain yield responses amongst different genotypes despite significant genotypic differences in resource allocation (e.g., harvest index, tiller number) and yield components in all experiments (e.g., grain number, ear number, kernel mass, and kernels per ear). Two cases were observed where certain yield components were impacted differently between cultivars. The unexpected reductions in ear number in Gregory at one defoliation time in Experiment 1; but these were compensated by increased kernels per ear so that grain number and grain yield was maintained. The only other case of differential responses to defoliation amongst genotypes was in Experiment 4, where the low harvest index of the undefoliated Scope barley was increased significantly by defoliation. The lack of interactions amongst the various yield components provides strong evidence that different genotypes responded very similarly to defoliation across these studies.

Previous defoliation studies have found that reductions in tiller number or ear number, which limit grain number and yield potential are often a key driver of yield reductions in defoliated crops (Kelman and Dove, 2009; Tian et al., 2012; Kirkegaard et al., 2015; Sprague et al., 2018). Genotypes with greater tillering capacity were thought to have greater plasticity in terms of recovering tillers after defoliation, to maintain grain number and yield, compared to genotypes with less tillering capacity (Kelman and Dove, 2009). However, across all experiments here, defoliation before GS30 did not reduce ear number significantly in any genotypes (except in Experiment 1, as discussed above). This is consistent with current understandings in winter wheats (Fieser et al., 2006; McMullen and Virgona, 2009; Harrison et al., 2011a) and is confirmed again here in spring cereals (Seymour et al., 2015). Experiment 4 tested genotypes with a wide range of tillering capacity (from 650 to 450 tillers/m^2^), and the high tillering barley produced twice as many ears as lower tillering wheat genotypes, yet there was no effect of defoliation on final ear number across any of these genotypes. A possible explanation is that defoliation well before GS30/31 is unlikely to remove or damage the main tillers and hence, later defoliations where this occurs may generate a greater response between genotypes varying in tilling capacity. In support of this, we only saw a reduction in main tiller numbers when defoliation occurred after GS30 (see Table 2), and early defoliation before GS 30 increased the total number of tillers per plant at anthesis in higher tillering cultivars (see Tables 2, 8). This increase in tiller production is likely due to increased light infiltration to the lower canopy after defoliation (Sparkes et al., 2006). Further, all the present experiments had no nitrogen or water stress during the period of tiller number determination, and hence the crop had sufficient resources to support the majority of tillers to maturity. It is plausible that combinations of water and/or nitrogen stress with defoliation may reduce assimilation sufficiently to reduce tiller survival during this critical period; this has not been examined here or by others to our knowledge.

Genotypic differences in accumulation of stem CHO prior to anthesis and translocation of these during grain filling is a trait associated with improved conversion of biomass to grain yield under terminal drought conditions (van Herwaarden et al., 1998). Severe defoliation during the vegetative phase can reduce the accumulation of these carbohydrates by removing biomass and reducing crop leaf area (Muir et al., 2006; Hu et al., 2019). Hence, reduction of CHO reserves could reduce the capacity of such genotypes to maintain grain yield after defoliation. However, the two experiments here included genotypes known to vary in this trait, but found no differences in their grain yield recovery after defoliation. We did not confirm the actual differences in CHO reserve accumulation between genotypes and how this may have been altered by defoliation, but further research may examine this. In the experiment under fully irrigated conditions (Experiment 3), the crops may not have been sufficiently source-limited after anthesis for previously stored CHOs to provide a significant benefit during grain filling. However, in this experiment kernel mass was reduced by defoliation (*P* = 0.08), which may indicate that defoliated crops were less able to fill the total grain sink. The varietal trait of accumulating CHO is known to offer greatest benefit under conditions of moisture stress during grain filling (van Herwaarden et al., 1998), and it is likely that there may be a strong seasonal interaction with defoliation reducing these reserves in wheat crops. While no response may be expected under irrigation (Experiment 3), a larger effect of CHO accumulating traits would be expected under rainfed conditions (Experiment 2). In experiment 2, while soil water was depleted quickly during grain filling, there was minimal moisture stress as the crops were still able to produce similar yields (>4.5 t/ha) and kernel mass as the fully irrigated experiment (Experiment 3). Hence, the full effects of stored CHO may not have expressed themselves under these conditions either. While CHO reserves may be reduced this may not actually reduce the total CHO that are translocated to grain during grain filling (Hu et al., 2019). Further, defoliation is known to slow the rate of water use leaving more soil water available at anthesis compared to the undefoliated crop (Harrison et al., 2011b); this was also observed in Experiment 2 (data not presented). This additional water available during grain filling is used more efficiently and is likely mitigating the effects of defoliation by offsetting any reductions in stored assimilates accumulated at anthesis.

Finally, we hypothesized that defoliation would have less effect on slower-developing genotypes with more time to recover leaf area and biomass than on faster-developing genotypes. However, we observed little evidence of this in these experiments although differences in development rate were relatively small. In Experiment 1, where we were able to reasonably synchronize flowering between the two cultivars, this amounted to a difference of <7 ± 3 days in the period between defoliation and anthesis between the fast and slower developing cultivars. These small differences are further confounded by difficulties in achieving synchronous development stages between different genotypes, meaning different genotypes are exposed to different environmental conditions. Here in Experiment 2, the two groups were sown too far apart (due to surface moisture conditions) or in Experiment 3 were sown on the same date, so key development phases did not coincide. Our results here add to many other studies that have found both different and similar responses to defoliation across wheat genotypes varying in their phenological development (Royo and Romagosa, 1996; Royo, 1997; McMullen and Virgona, 2009; Sprague et al., 2018). This lack of consistency suggests this is a problematic relationship to unravel experimentally as it is very difficult to isolate the environmental conditions from genotypic effects and their interactions. An appropriately characterized crop growth model which integrates both regrowth and phenological effects on the crop may be able to add deeper understandings on how defoliation intensity and timing may influence the capacity of different genotypes to compensate. While others have attempted to model the trade-offs between grazing and grain yield in wheat crops (Zhang et al., 2008; Harrison et al., 2012), these models have not mechanistically captured the phenological development changes and how this would interact with environmental or genotypic differences in cultivars. Characterizing the physiological processes driving regrowth after defoliation in wheat (and other crops) is possible in models like APSIM (Holzworth et al., 2014) and hence examining interactions of genotype with water and nitrogen availability and grazing management would inform further experimental work and/or better inform agronomic recommendations.

### Defoliation Effects on Crop Regrowth and Yield

Across all experiments, when crops were defoliated before GS30 yield penalties were less than 0.7 t/ha and relative yields (% of undefoliated crops) were greater than 85%; the average yield penalty across all experimental treatments was 0.36 t/ha. These yield penalties are like other studies where cereal crops are grazed or defoliated prior to stem elongation (Edwards et al., 2011; Harrison et al., 2011a; Frischke et al., 2015; Seymour et al., 2015). Larger yield penalties were observed in Experiment 1 where severe defoliation occurred after GS30, as has been reported by others (Harrison et al., 2011a). Defoliation reduced biomass at anthesis by 1-2 t DM/ha (20% reduction on average) in almost every genotype (excluding Hindmarsh barley in Experiment 4). Maturity biomass was reduced by a similar magnitude. These reductions were typically much larger than the removal of biomass by defoliation in each experiment, showing that there is an extended influence of slower plant growth after defoliation, associated with lower leaf area and accumulated radiation interception (Harrison et al., 2011c). Defoliated crops also often had a reduced LAI and Ri at anthesis; however, this was not universal across all experiments and treatments. While maximizing radiation interception at anthesis is regarded as critical to maximize grain number (Fischer, 1985), here we only observed a significant reduction in grain number (and hence grain yield) when Ri was reduced by more than 0.2 after later defoliation treatments. Most previous studies have observed reductions in kernel number after defoliation, associated with lower final ear number and/or reduced kernels per ear. Crops able to more efficiently fill this sink can compensate to maintain grain yield. In contrast, defoliation resulted in a reduced kernel mass in 3 of the 4 experiments reported here (all irrigated), while grain number was unaffected. In other studies, increases in crop harvest index have been reported after defoliation, which is typically influenced by undefoliated crops having a low conversion of biomass into grain, often associated with post-anthesis water stress. The lack of moisture stress in the present experiments may have enabled undefoliated crops to effectively fill their grain sink and defoliated crops were unable to ‘catch-up’ due to lower leaf area and biomass at the start of grain filling. This is further supported by calculations of the ratio of grain yield to post anthesis growth, where the undefoliated wheat crops were always higher (average of 1.37) than the defoliated wheat crops (average of 0.93), meaning that yield of the defoliated crops were more reliant on growth potential after anthesis. Together these data suggest that under plentiful water and nitrogen supply, the reduction in leaf area and biomass after defoliation is likely to have a detrimental effect on post-anthesis growth potential, while under stressful post-anthesis conditions, defoliated crops are more likely to be able to compensate. Experiments where water supply is manipulated to induce stress in combination with defoliation would help further our understanding of these interactions.

### Potential for Dual-Purpose Use of Spring Cereals

This research shows that spring cereals can offer potential as dual-purpose graze and grain crops in growing environments with a short growing season (e.g., <5 months) where longer duration cultivars are unsuited (Bell et al., 2015b). In all experiments here the crops provided small but valuable amounts of high-quality forage (0.3–1.2 t DM/ha) before stem elongation, with limited risks of substantial yield reductions. These levels of biomass available were similar to those measured in lower rainfall environments in southern Australia (Frischke et al., 2015; Latta, 2015; Seymour et al., 2015). Based on an allowance of 1.5 kg of biomass per sheep per day this translates into 200–800 DSE grazing days/ha, which is consistent with the predictions of grazing from spring wheats using APSIM (Bell et al., 2015b). This translates into an additional AU$ 120–480/ha of income that can be obtained by grazing (assuming $2/kg LW and 0.3 kg LW/d when grazing wheat). This income from grazing is sufficient to offset yield reductions of 0.5 to 2.0 t grain/ha (assuming AU$240/t of wheat), which is more than the yield penalties for any of the defoliation treatments implemented here except when defoliated after GS30 (Experiment 1). Further, this lack of yield penalties was despite most of these experiments being managed under fully irrigated conditions, where post-anthesis moisture stress did not occur to reduce the harvest index of the undefoliated crops relative to the defoliated crops. In conditions with terminal drought where defoliation may help with slowing soil water use until after anthesis, yield reductions are likely to be smaller. Defoliation may actually increase grain yield, particularly in systems where crops are grown on stored soil moisture (e.g., subtropical regions) and delaying its use until post anthesis can greatly enhance efficiency of grain fill (Zhu et al., 2004). Despite the potential shown here, the defoliation in our experiments was implemented mechanically and other yield reducing factors (e.g., plant trampling, plant removal or soil surface compaction) may impact further on the crops ability to recover yield, although in cases where grazing and mechanical defoliation have been compared there has been found to be little difference (Pumphrey, 1970; Francia et al., 2006; Harrison et al., 2011a).

Longer season wheat cultivars sown earlier also provided more biomass by GS 30, however, there was little difference in biomass accumulation amongst cultivars where they were sown and defoliated at the same time. These results are consistent with model predictions of grazing potential from different spring wheat cultivars across environments, where slower spring cultivars (e.g., Gregory) can be sown slightly earlier and provide more grazing potential than later sown fast spring cultivars (e.g., Crusader, H45) (Bell et al., 2015b). Similar to other studies we also found that barley has a higher vegetative biomass production potential than wheat when sown at the same time (Francia et al., 2006; Latta, 2015; Sprague et al., 2018). Further, barley grain value is often lower than wheat, so less grazing is needed to offset any potential yield penalties. Hence, barley may provide a preferable dual-purpose option in shorter-growing season environments with limited capacity to sow earlier to allow for a longer grazing period.

## Conclusion

Differences in physiological attributes of cereal cultivars were found to have little influence on the capacity of spring wheat to recover after defoliation. Hence, amongst genotypes with similar fast phenological development that are bred and grown primarily for grain yield attributes there seems to be little practical difference in their capacity to recover after defoliation. Other research has shown that longer-season winter cultivars developed for dual-purpose use are likely to provide greater grazing potential and have longer time to recover enough leaf area and biomass to achieve similar grain yields and hence cultivar selection may be more important (Carver et al., 2001; Thapa et al., 2010). Our research demonstrates that even in fast developing spring cultivars in warm growing environments, opportunistic removal of small amounts of biomass prior to stem elongation (GS31) can be achieved without significant reductions in grain yield. Further research should focus more on grazing management or defoliation timing and intensity before this critical point, to explore how these interact with crop recovery, rather than testing a range of cultivars under inconsistent management. Providing more rigorous guidelines and tools for farmers to make decisions about when to stop grazing or how much biomass to retain during grazing will minimize the risk of yield penalties from grazing and enhance the dual-purpose use of crops across a range of environments. In particular, understanding the residual biomass and time required to recover enough biomass and/or leaf area prior to anthesis to mitigate potential losses in grain number which is well known to be the main effect of defoliation (Royo and Romagosa, 1996; Edwards et al., 2011; Harrison et al., 2011b; Tian et al., 2012). This research clearly shows that in addition to the slower-developing winter-type genotypes widely used for grazing, the faster-developing spring cultivars can also be safely grazed. However, more effort is needed to understand if more diverse phenology types (e.g., winter vs. spring types) require different grazing recommendations.

## Data Availability Statement

The datasets generated for this study are available on request to the corresponding author.

## Author Contributions

LB coordinated and implemented the experiments, wrote the manuscript, and conducted the data analysis. JK inputs into experimental design, edited the manuscript, and led the project. LT implemented one of the experiments and conducted the data analysis. SM implemented one of the experiments and conducted the data analysis. JL contributed to implementing three of experiments.

## Conflict of Interest

The authors declare that this study received funding from the Grains Research and Development Corporation, Australia. The funder was not involved in the study design, collection, analysis, interpretation of data, the writing of this article or the decision to submit it for publication.
